# Identification of Candidate Genes for Min Pig Villi Hair Traits by Genome-Wide Association of Copy Number Variation

**DOI:** 10.3390/vetsci10050307

**Published:** 2023-04-23

**Authors:** Xinmiao He, Ming Tian, Wentao Wang, Yanzhong Feng, Zhongqiu Li, Jiahui Wang, Yan Song, Jinfeng Zhang, Di Liu

**Affiliations:** 1 Institute of Animal Husbandry, Heilongjiang Academy of Agricultural Sciences, Harbin 150086, Chinatianming@haas.cn (M.T.); wangwentao_1981@163.com (W.W.); lixiang@haas.cn (Y.F.); lizhongqiu1974@163.com (Z.L.); 2 Branch of Animal Husbandry and Veterinary of Heilongjiang Academy of Agricultural Sciences, Qiqihar 161005, China; vip13895994813@163.com (J.W.);; 3 Harbin Academy of Agricultural Sciences, Harbin 150029, China

**Keywords:** Min pig, villi hair follicle, copy number variation, genome-wide association study

## Abstract

**Simple Summary:**

Min pigs living in northeast China have the characteristics of good meat quality, and strong disease- and cold-resistance characters. Min pig is also one of the few pig breeds with villi hair in the world. The study of its villi hair traits is of great significance for the mechanism of cold resistance and animal welfare. In this study, a Large White × Min pigs F2 population was constructed, and a case-control genome-wide association study was performed to investigate the potential copy number variation (CNVs) associated with villi hair appearance. Finally, we found important genes related to villi. This study may also provide a basic reference for the selection and breeding of cold-resistant pigs and outdoor breeding.

**Abstract:**

The Min pig is a famous native pig breed in northeast China, which has the special genetic character of villi hair growth in cold seasons. At present, little research has focused on the genetic mechanism of villi hair growth in Min pigs. Copy number variations (CNVs) are a type of variant that may influence many traits. In this study, we first investigated the phenotype of Large White × Min pigs’ F2 pig villi hair in detail and then performed a CNV-based genome-wide association study (GWAS) between CNVs and pig villi hair appearance. Finally, a total number of 15 significant CNVRs were found to be associated with Min pig villi hair. The most significant CNVR was located on chromosome 1. Nearest gene annotation analysis indicated that the pig villi hair traits may be associated with the biological process of the G-protein-coupled receptor signaling pathway. QTL overlapping analysis found that among the CNVRs, 14 CNVRs could be co-located with known QTLs. Some genes such as *MCHR2*, *LTBP2*, and *GFRA2* may be candidate genes for pig villi traits and are worth further study. Our study may provide a basic reference for the selection and breeding of cold-resistant pigs and outdoor breeding.

## 1. Introduction

In the long-term evolution of mammals, many species have undergone environmental adaptation, such as for cold, rather than geographic migration [1]. As a result, the fur or hair of many cold-resistant animals presents seasonal growth characteristics, such as villi hair growth in winter [2]. Until now, in the world, during the long-term breeding process, domestic pigs have gradually eliminated seasonal growth of villi hair and only a small number of Chinese native pigs such as the Min pig were exceptions which showed the characteristics of cold adaptation.

In mammals, the hair follicles mostly developed circularly, and the villi hair was produced by secondary hair follicles [3]. In previous reports, research on human and mouse hair growth was mainly focused on related diseases, such as alopecia and leukotrichia [4]. In economically fur-bearing animals, such as cashmere goats, yaks, and mink, research was mainly focused on hair follicle growth [5]. There are only a few reports concerning the genetics of animal villus hair, and candidate genes related to hair development was plakophilin 1 (*PKP1*), forkhead box family genes, keratins (KRTs), *chi*-*miR*-*30e*-*5p*, and delta-like canonical notch ligand 4 (*DLL4*) [3,6,7,8].

Copy number variations (CNVs) are one of the variation types with wide coverage of the genome which are most likely to explain the “missing inheritance” [9]. Analysis of the association between villi hair traits and CNVs may also be useful for the research of the villi hair development mechanism. In this study, we used a CNV-based genome-wide association study (GWAS) to analyze the unique villi hair growth of Min pigs through the determination of villi hair appearance and CNV genotype of the pigs. We aimed to explore the potential relationship between genes and villi growth. Our findings may provide some reference for animal welfare or for the selection and breeding of cold-resistant pigs and outdoor breeding.

## 2. Materials and Methods

### 2.1. Ethics Statement

All animals used in this study were raised in the experiment base of the Institute of Animal Science, Chinese Academy of Agricultural Sciences (IAS, CAAS) (Beijing, China) and treated according to the guidelines for experimental animal welfare and ethics of IAS, CAAS (IAS2020-109). Animal tissue collections were also approved by the Science Research Department of the IAS, CAAS (Beijing, China).

### 2.2. Animals and Phenotype Recording

We recorded the presence or absence of villi hair in the necks of pigs (older than 3 months old) in late autumn (after 1 October), winter, and early spring (1 March) from a Large white pig × Min F2 resource population. The presence of villi hair was recorded as “1” and the absence was recorded as “0”.

### 2.3. Genotyping and Quality Control

DNA of ear tissue for the F2 individuals (573 individuals) and F0 individuals (19 individuals) was extracted using the phenol–chloroform method. After next-generation sequencing of the population, we used CNVcaller software (version 0.11) to investigate and to genotype all individual CNVs. All the details of resequencing, CNV calling, and genotyping were the same as described in the study of Wei et al. [10].

### 2.4. Genome-Wide Association Study

We conducted case-control association tests to identify a significant association between villi hair appearance and genome wide significant CNVRs using PLINK (www.cog-genomics.org/plink2 (accessed on 16 Jan 2023)). The genome-wide significance threshold was determined to be 9.71 × 10^−5^ (0.05/515), and the chromosome-wide significance threshold was 1.94 × 10^−3^ (1/515) using the Bonferroni method [11], where 515 is the number of informative CNVRs after quality control in the dataset.

### 2.5. Gene Enrichment Analysis and QTL Co-Location

Gene ontology (GO) terms and Kyoto encyclopedia of genes and genomes (KEGG) analysis were all performed using KOBAS 3.0 software (Beijing, China), with a significant threshold of *p*-value < 0.05. For the analysis of CNVRs and QTLs co-location, QTLs were first retrieved from the PigQTLdb (https://www.animalgenome.org/cgi-bin/QTLdb/SS/index (accessed on 28 December 2022)) [12] and then overlapped with CNVRs using Bedtools (v. 2.27.1) software [13] with the command line of: intersectBed -a -b -wa-wb > QTLs.txt.

### 2.6. Validation of CNVs by Real-Time Quantitative PCR (qRT-PCR)

For each validated CNVR, we select ten individuals (five individuals in each CNVR genotype group) for qRT-PCR amplification to confirm the CNV calling and genotyping results. We use Primer Premier 6.0 software (Biosoft International, Palo Alto, CA, USA) to design the primers of validate CNVRs. The glucagon gene (GCG) was used as the single-copy control. Four CNVRs, which were CNV13, CNV667, CNV540, and CNV64, were compared independently between the normal type and Duplicate/Deleted type using the simple t-test in SAS software version 9.2 (SAS Institute, Inc., Cary, NC, USA). The primer sequences used in this study are shown in Table 1.

## 3. Results

### 3.1. Phenotype Description

In the population of 573 Min × Large white F2 pigs, we found the phenomenon of significant trait segregation, and the neck villi hair was found in 198 F2 individuals (recorded as “1”), and not found in 375 F2 individuals (recorded as “0”), respectively. We inferred that the appearance of villi hair may be a classic dominant–recessive inheritance trait. However, as we did not record the phenotype of F1 pigs, we could not confirm the conclusion. Moreover, we also found that the villi hair appearance has no relationship with the coat color of F2 pigs.

### 3.2. Copy Number Variation Genotyping

**Figure 1:**
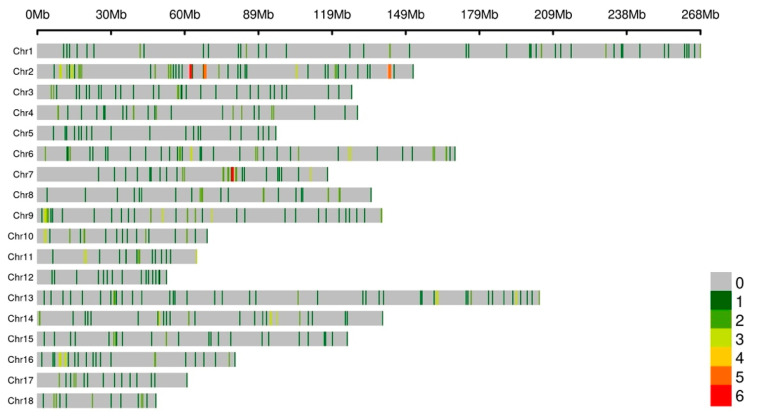
The genomic distribution of pig CNV regions (CNVRs). Different colors of the line within the chromosome indicate different number of CNVRs in the 1 Mb size region.

In both F2 and F0 population pigs, CNV regions (CNVRs) were called and genotyped. Finally, a number of 1090 CNVRs were identified, including 370 duplicated-type (Dup) of CNVRs, 412 deleted-type (Del) of CNVRs. There are also 308 CNVRs with both duplicated and deleted status (Figure 1). The average length of CNVRs on chromosomes 4, 5, 15, and 16 were shorter than on other chromosomes.

### 3.3. GWAS of Pig Villi Hair

**Figure 2:**
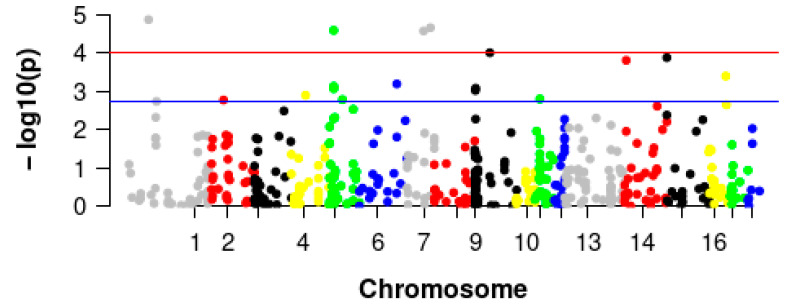
Manhattan plot of associated CNVs for villi hair appearance trait. The blue line indicates chromosome significant threshold, and the red line indicates the genome-wide significance threshold. Different colored dots indicate different chromosomes.

After rigorous quality control, a number of 490 pigs and only 515 CNVRs were finally screened to use in the GWAS analysis between villi hair appearance traits and CNVRs. A total of 15 CNVRs were detected significantly associated with villi hair traits using Plink software (Table 2 and Figure 2). In all, 10 of the 15 CNVRs were duplication and normal type, and 5 of the 15 CNVRs were deletion and normal type.

The 15 significant associated CNVRs were distributed on 11 chromosomes. On chromosome 1, a total of two CNVRs were detected significantly associated with villi hair appearance (Table 2). The strongest association of these CNVRs was CNV13. CNV13 is located near the gene of activating signal cointegrator 1 complex subunit 3 (*ASCC3*), melanin concentrating hormone receptor 2 (*MCHR2*), and single-minded family BHLH transcription factor 1 (*SIM1*). In the F0 generation, all of the Large white pig CNV13 genotypes were deletion, and all of the Min pig CNV13 genotypes were normal (none duplicated or deleted).

On chromosome 5 and chromosome 7, there were also two CNVRs detected significantly associated with villi hair appearance, respectively. Furthermore, the second- and third-strongest associated CNVRs (CNV540 and CNV64) were on chromosome 5 and chromosome 7 respectively. Only one significant CNVR was located on chromosomes 2, 4, 6, 11, 14, 15, and 16, respectively, and a total of 73 known genes were found in the up and down 50k stream sequences.

### 3.4. Identification and Validation of the Selected CNVRs

**Figure 3:**
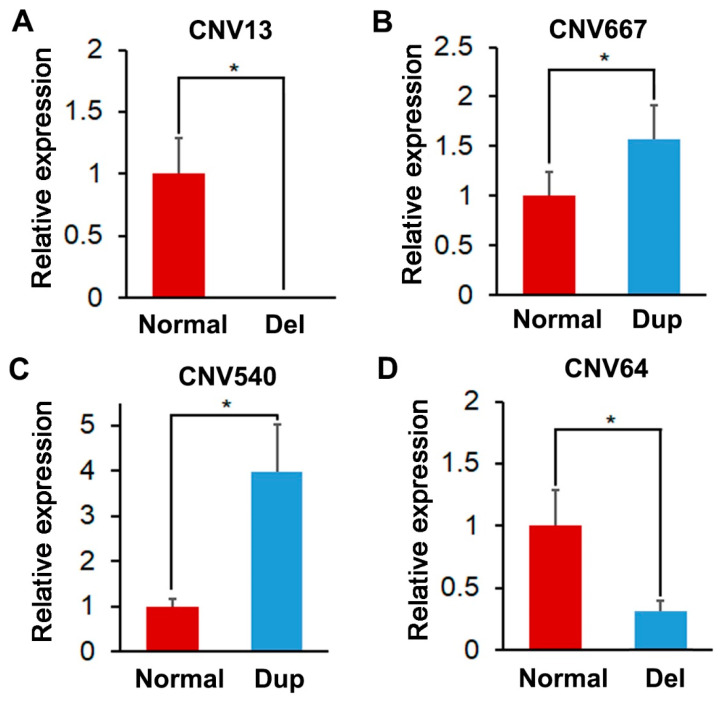
Relative expression of selected CNVRs (**A**) Relative expression of normal and deletion (Del) type of CNV13; (**B**) Relative expression of normal and duplicated (Dup) type of CNV667; (**C**) Relative expression of normal and Dup type of CNV540; (**D**) Relative expression of normal and deletion (Del) type of CNV64. A * represents significant difference (*p* < 0.05).

In this study, we used the DNA qRT-PCR validation method to confirm the validity and truth of investigated CNVRs. The first four significant CNVRs were selected for validation. The relative expression results were shown in Figure 3A–D. As shown, one hundred percent (4/4) of the selected CNVRs could be validated by DNA qRT-PCR.

### 3.5. Functional Enrichment and Annotation Analysis

**Figure 4:**
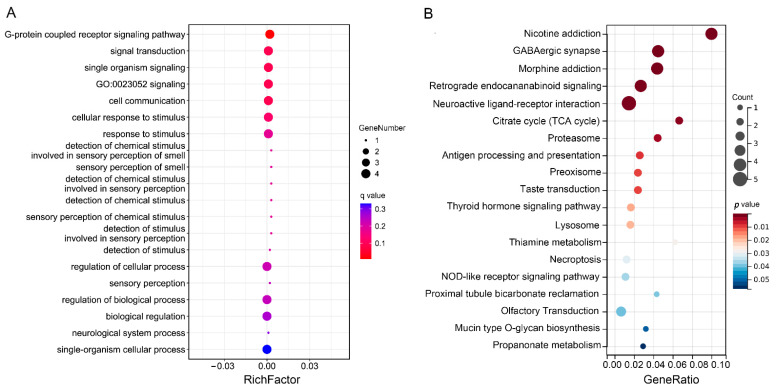
Enrichment of significant CNVRs’ nearest genes. (**A**) Top 20 GO enrichment terms of biological process for CNVRs nearby genes. (**B**) Top 20 KEGG Pathways of CNVRs nearby genes.

As CNVRs may play their role in combination with nearby genes, or the CNVRs may be one of the linkage signals with nearby SNPs, we analyze the function of CNVRs’ nearby genes in 50K up and down stream sequences. The GO enrichment showed that the relative genes were mainly involved in the biological process of G-protein coupled receptor signaling pathway, signal transduction, single organism signaling, cell communication, and so on (Figure 4A). The KEGG enrichment showed that the relative genes were mainly involved in the pathways of GABAergic synapse, NOD-like receptor signaling pathway, and so on (Figure 4B).

**Figure 5:**
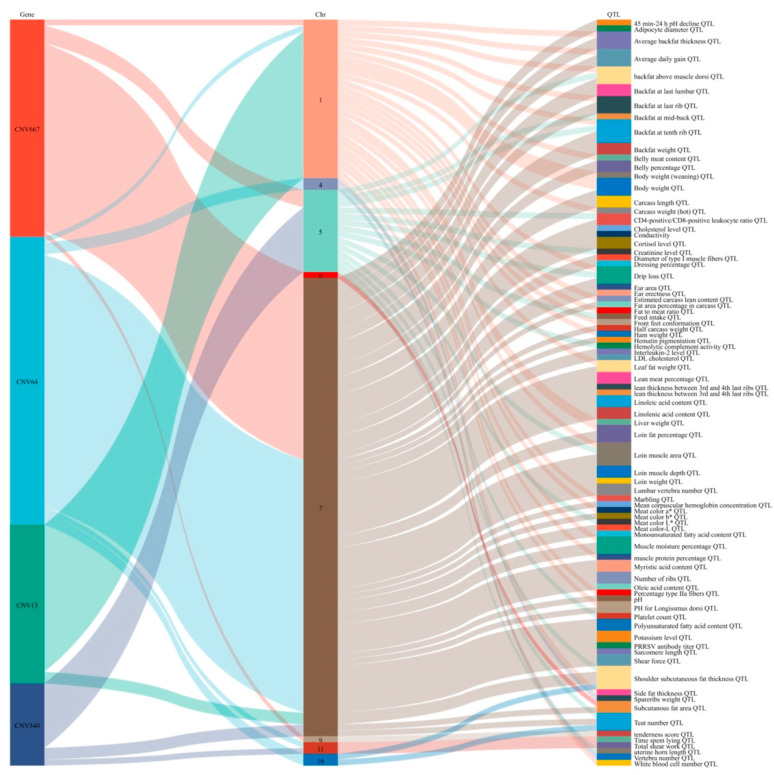
The intersection of the 4 most significant CNVRs with swine QTL data. The first column was the name of CNVRs, the second column was the name of chromosome, the third column was name of QTLs.

In order to screen the potential function of the CNVRs, we also compared the 15 significant CNVRs with QTLs in the pig QTLdb and previous reports of the economic traits. The results were shown in Figure 5 and supplementary Figures S1–S3. Overall, 14 of the 15 significant CNVRs could overlap with at least one trait. As no research has reported the genes or QTLs with villi hair, none of the CNVRs could be overlapped with the villi hair appearance trait.

## 4. Discussion

In previous studies, the genetic characteristics of Min pigs were mainly studied from different traits such as disease resistance, meat quality, and so on [14,15]. In our previous study, we sequenced the mRNA transcriptome of Min pig hair follicles and screened some candidate genes which may affect villi hair [6]. As the villi hair appeared only in Min pigs but none in Large white pigs, the Min × Large white F2 resource population was an ideal population for investigating the candidate genes with villi hair. In this study, we first used next-generation sequencing data of the Min × Large white F2 resource population for CNV calling and genotyping, and then performed CNV-based GWAS for villi hair trait candidate CNV identification. This will help us not only to investigate candidate genes but also to better understand the potential genetics mechanism of villi hair growth in Min pigs. Finally, we investigated 15 CNVRs which may have potential affection with villi hair.

The most significant villi-hair-associated CNVR we investigated was CNV13. In order to confirm the function of CNV13, we detected the genotype of CNV13 in the F0 population pigs. We found that the normal copy number of CNV13 was only apparent in the pure Min pigs, and the deletion status of CNV13 only appeared in the pure Large White pigs, and this result is consistent with the phenotype of villi hair appearance trait of these two breeds. As the sequencing depth was 30× in the F0 population, the existence of CNV13 was confirmed. Moreover, in order to identify the potential function of significant CNVRs, the function of the known genes in which significant CNVRs were located was retrieved.

The nearest gene around CNV13 was *MCHR2*. In previous research, *MCHR2* was reported encoding one of two G-protein coupled receptors for melanin-concentrating hormone (MCH), which is involved in energy homeostasis [16,17], and a neuropeptide synthesized in the region of the brain critical for feeding and reward [18,19]. There is also a CNV presented in *MCHR2* promoter region. In the research of Fischer et al., they performed a genome-wide CNV analysis in a 585 alopecia areata patients and 1340 controls in a European population, and the result indicated that the CNV in *MCHR2* promoter may associate with alopecia areata [20]. We inferred that, maybe in pigs, CNV13 has strong linkage with CNV in *MCHR2* or has interaction with *MCHR2*, and finally affects the hair development. As no CNV was detected in the *MCHR2* gene in our population, we inferred that the interaction between CNVRs and *MCHR2* may be more possible. In any case, more experiments and analysis such as gene interaction analysis and the relative expression of *MCHR2* of different CNV13 genotype analysis should be conducted.

The second significant villi-hair-associated CNVR was CNV667. This region was a famous region with many traits, such as number of ribs. There are 11 genes around this CNVR within 50K distance, including latent TGF-beta binding protein2 (*LTBP2*) and homozygous mutation in ATP Binding cassette subfamily D member 4 (*ABCD4*). The major function of latent TGF-β binding proteins (LTBPs) is modulating transforming growth factor beta (TGF-β) activity and finally controls growth factor secretion, matrix deposition, presentation, and activation [21]. In the report of Wollina et al., TGF-β could be involved in the initiation of catagen for the follicle epithelium as well as growth control for sebaceous glands [22]. *ABCD4* could encode a member of the superfamily of ATP-binding cassette (ABC) transporters [23]. In the research of Takeichi et al., *ABCD4* has been reported to play a role in the intracellular processing of cobalamin and finally induced a slight lightening of her previously black hair [24]. As the villi hair growth may associate with the process of skin and follicle development, we inferred that these two genes may associate with villi hair trait and need to be further researched.

Around CNV234, there is also one notable gene, named Glial cell line-derived neurotrophic factor receptor alpha 2 (*GFRA2*). *GFRA2* is a receptor for glial-cell-- line-derived neurotrophic factors, which are survival factors that regulate the differentiation of many peripheral neurons [25]. In previous research, *GFRA2* was not only reported to influence the basal metabolic rates, but also reported to be involved in the control of hair-follicle cycling [26,27]. Around CNV330 on chromosome 6, the GIPC PDZ domain containing family member 2 (*GIPC2*), which could activate WNT signaling, also indicates villi hair regulation potential. In the research of Bhat et al., *GIPC2* was more significantly expressed (*p* < 0.05) in the hair follicle telogen phase than in the hair follicle anagen phase [28]. Moreover, these two genes also require further research to confirm their function in hair development.

Another notable gene was Nudix hydrolase 15 (*NUDT15*) which is located near CNV107 on chromosome 11. The gene of *NUDT15* could encode an enzyme which is a negative regulator of thiopurine activation and toxicity [29], and a *NUDT15* R139C mutation could cause thiopurine-induced early hair loss in Japanese and Korean patients [30,31]. Moreover, *NUDT 15* c.415C > T polymorphism could also increase the risk of hair loss, as pointed out in the research of Wang et al. [32].

Moreover, there also some genes associated with malignant skin tumors, such as the gene of proteasome activator subunit 1 (*PSME1*) [33], proteasome activator subunit 2 (*PSME2)* [34], which is located near the fourth significant CNVR-CNV64, and docking protein 2 (*DOK2*) [35], which is located near CNV234 on chromosome 14. As we know, the villi hair development is partly a result of adaptation to cold (or hot) climates, so, we searched all of the genes near or within the 15 CNVRs with cold or hot adaptation. Among these genes, activating transcription factor 1 (*ATF1*) could modulate the heat shock response, and was related to thermal adaptations in cattle [36,37]. In the report of Saravanan et al., dnaJ heat shock protein family (Hsp40) member B4 (*DNAJB4*) was also a candidate gene that may be related to heat stress in cattle [38]. In the report of Ai et al. the gene of *SIM1* is likely important for genetic adaptation to high altitude (cold and hypoxia) in pig [39]. These genes may also be involved in the hair development, but more evidence needs to be explored.

## 5. Conclusions

The Min pig is an ideal animal model to study villi hair development. Through GWAS of villi hair traits in a F2 population, a total of 15 CNVRs were found to be significantly associated with pig villi hair appearance. The pig villi hair traits may be associated with the biological process of the G-protein coupled receptor signaling pathway and so on. Some genes such as *MCHR2*, *LTBP2*, *GFRA2*, *GIPC2*, and *NUDT15* may be candidate genes for the pig villi trait and are worth further study. Our study may provide a basic reference for the selection and breeding of cold-resistant pigs and outdoor breeding.

## Figures and Tables

**Table 1:** Primers for the selected CNVRs and GCG.

Gene	Forward Primer	Reverse Primer
CNV13	CCTGGCTTCCCTTCTCTCTT	GGAAGAATCGGGTGGGTATT
CNV667	AGCTTCCTGCTGGACACAGT	GCTGCTTTGGGCTTATTCTG
CNV540	CTTCAAAGCCAGCAATGTGA	GGATTATCCGAGTTGGCTCA
CNV64	TCTGAAAGGGATGGATCTGG	GAACCTTTGCTGCCTGTCTC
GCG	GCAATATGGCTTTAGAATACACCTCTTA	GTCACTAATCAAGATCGTGTTCACAAC

**Table 2:** Descriptions of the significant CNVRs associated with villi hair appearance trait.

CNVR ID	Location	Nearest Genes	CHISQ	Type	*p*-Value
CNV13	1:67141601-67155200	*ASCC3*/*MCHR2*/*SIM1*	18.9	Del	1.38 × 10^−5^
CNV667	7:97857601-97860000	*VSX2*/*ABCD4*/*VRTN*/*SYNDIG1L*/*ISCA2*/*LTBP2*/*NPC2*/*AREL1*/*FCF1*/*YLPM1*/*PROX2*	17.97	Dup	2.24 × 10^−5^
CNV540	5:16751601-16754400	*DIP2B*/*ATF1*/*TMPRSS12*/*SLC4A8*/*SCN8A*	17.66	Dup	2.64 × 10^−5^
CNV64	7:75399601-75402400	*REC8*/*IRF9*/*FITM1*/*PSME1*/*RNF31*/*PSME2*/*EMC9*/*DCAF11*/*CARMIL3*/*CPNE6*/*PCK2*/*NRL*/*DHRS4*/*ZFHX2*/*JPH4*/*AP1G2*/*THTPA*/*MYH7*/*ENSSSCG00000002020*	17.6	Del	2.73 × 10^−5^
CNV734	9:51430801-51434000	*OR8D1*/*OR8D2*/*ENSSSCG00000015179*/*ENSSSCG00000061653*/*ENSSSCG00000053222*/*ENSSSCG00000015193*/*ENSSSCG00000053475*/*OR8B8*/*ENSSSCG00000056190*/*ENSSSCG00000055348*/*ENSSSCG00000061394*/*ENSSSCG00000015154*/*ENSSSCG00000052681*	15.12	Del	0.000101
CNV267	15:1259201-1261200	*ENSSSCG00000060497*/*NMI*/*TNFAIP6*	14.56	Del	0.000135
CNV234	14:6050401-6053200	*ENSSSCG00000055110*/*GFRA2*/*XPO7*/*DOK2*/*DMTN*/*FGF17*	14.26	Dup	0.000159
CNV321	16:61828001-61833200	*GABRG2*/*GABRA1*/*GABRA6*/*GABRB2*	12.49	Dup	0.00041
CNV330	6:135400401-135405200	*FUBP1*/*DNAJB4*/*NEXN*/*MIGA1*/*USP33*/*GIPC2*/*ENSSSCG00000035986*/*ENSSSCG00000050806*	11.61	Del	0.000657
CNV720	9:4729201-4731600	*ENSSSCG00000060217*/*ENSSSCG00000062640*/*ENSSSCG00000050624*/*ssc*-*mir*-*10390*	11.13	Dup	0.000849
CNV510	4:47520801-47538800	*ENSSSCG00000042115*	10.35	Dup	0.001295
CNV107	11:19803201-19810400	*ENSSSCG00000042466*/*SUCLA2*/*NUDT15*/*MED4*/*ENSSSCG00000053915*	9.936	Dup	0.001621
CNV552	5:45503601-45508000	*CCDC91*/*ENSSSCG00000045661*/*PTHLH*	9.868	Dup	0.001682
CNV390	2:47668801-47674000	*NSSSCG00000031143*/*USP47*/*DKK3*/*U6*/*GALNT18*	9.819	Dup	0.001727
CNV22	1:93250401-93252800	*ENSSSCG00000005790*/*ENSSSCG00000053538*	9.671	Dup	0.001872

CNVR: copy number variation region; Dup: duplicate; Del: deletion; CHISQ: chi square test; *ASCC3*: activating signal cointegrator 1 complex subunit 3; *MCHR2*: melanin concentrating hormone receptor 2; *SIM1*: SIM BHLH transcription factor 1; *VSX2*: visual system homeobox 2; *ABCD4*: ATP binding cassette subfamily D member; *VRTN*: vertebrae development associated; *SYNDIG1L*: synapse differentiation inducing 1 like; *ISCA2*: iron-sulfur cluster assembly 2; *LTBP2*: latent transforming growth factor beta binding protein 2; *NPC2*: NPC intracellular cholesterol transporter 2; *AREL1*: apoptosis resistant E3 ubiquitin protein ligase 1; *FCF1*: FCF1 RRNA-Processing protein; *YLPM1*: YLP motif containing 1; *PROX2*: prospero homeobox 2; *DIP2B*: disco interacting protein 2 homolog B; *ATF1*: activating transcription factor 1; *TMPRSS12*: transmembrane serine protease 12; *SLC4A8*: solute carrier family 4 member 8; *SCN8A*: sodium voltage-gated channel alpha subunit 8; *REC8*: REC8 meiotic recombination protein; *IRF9*: interferon regulatory factor 9; *FITM1*: fat storage inducing transmembrane protein 1; *PSME1*: proteasome activator subunit 1; *RNF31*: ring finger protein 31; *PSME2*: proteasome activator subunit 2; *EMC9*: ER membrane protein complex subunit 9; *DCAF11*: DDB1 and CUL4 associated factor 11; *CARMIL3*: capping protein regulator and myosin 1 linker 3; CPNE6: copine 6; *PCK2*: phosphoenolpyruvate carboxykinase 2, mitochondrial; *NRL*: neural retina leucine zipper; *DHRS4*: dehydrogenase/reductase 4; *ZFHX2*: zinc finger homeobox 2; *JPH4*: junctophilin 4; *AP1G2*: adaptor related protein complex 1 subunit gamma 2; *THTPA*: thiamine triphosphatase; *MYH7:* myosin heavy chain 7; *OR8D1*: olfactory receptor family 8 subfamily D member 1; *OR8D2*: olfactory receptor family 8 subfamily D member 2; *OR8B8*: olfactory receptor family 8 subfamily B member 8; *NMI*: N-Myc and STAT interactor; *TNFAIP6*: TNF alpha induced protein 6; *GFRA2*: GDNF family receptor alpha 2; *XPO7*: exportin 7; *DOK2*: docking protein 2; *DMTN*: dematin actin binding protein; *FGF17*: fibroblast growth factor 17; *GABRG2*: gamma-aminobutyric acid type A receptor subunit gamma 2; *GABRA1*: gamma-aminobutyric acid type A receptor subunit alpha 1; *GABRA6*: gamma-aminobutyric acid type A receptor subunit alpha 6; *GABRB2*: gamma-aminobutyric acid type A receptor subunit beta 2; *FUBP*: far upstream element binding protein; *DNAJB4*: dnaJ heat shock protein family (Hsp40) member B4; *NEXN*: nexilin f-actin binding protein; *MIGA1*: mitoguardin 1; *USP33*: ubiquitin specific peptidase 33; *GIPC2*: GIPC PDZ domain containing family member 2; *SUCLA2*: succinate-CoA ligase ADP-forming subunit beta; *NUDT15*: nudix hydrolase 15; *MED4*: mediator complex subunit 4; *CCDC91*: Coiled-Coil domain containing 91; *PTHLH*: parathyroid hormone like hormone; *USP47*: ubiquitin specific peptidase 47; *DKK3*: dickkopf WNT signaling pathway inhibitor 3; *GALNT18*: polypeptide N-Acetylgalactosaminyltransferase 18.

## Data Availability

The datasets presented in this study can be found in online repositories. The sequencing data used in the current study have been submitted to the Genome Sequence Archive, with the accession number CRA002451.

## Supplementary Materials

The following supporting information can be downloaded at: https://www.mdpi.com/article/10.3390/vetsci10050307/s1, Figure S1: The intersection of CNV510, CNV552, and CNV734 with swine QTL data; Figure S2: The intersection of CNV 22, CNV267, and CNV390 with swine QTL data; Figure S3: The intersection of CNV321, CNV330, CNV720 and CNV107 with swine QTL data.
