# Supplementary material for: Identification of Candidate Genes for Min Pig Villi Hair Traits by Genome-Wide Association of Copy Number Variation
Source: Vet Sci. 2023 Apr 23;10(5):307. doi: 10.3390/vetsci10050307 (PMC10222405; doi:10.3390/vetsci10050307)

Figure S1: The intersection of CNV510, CNV552, and CNV734 with swine QTL data

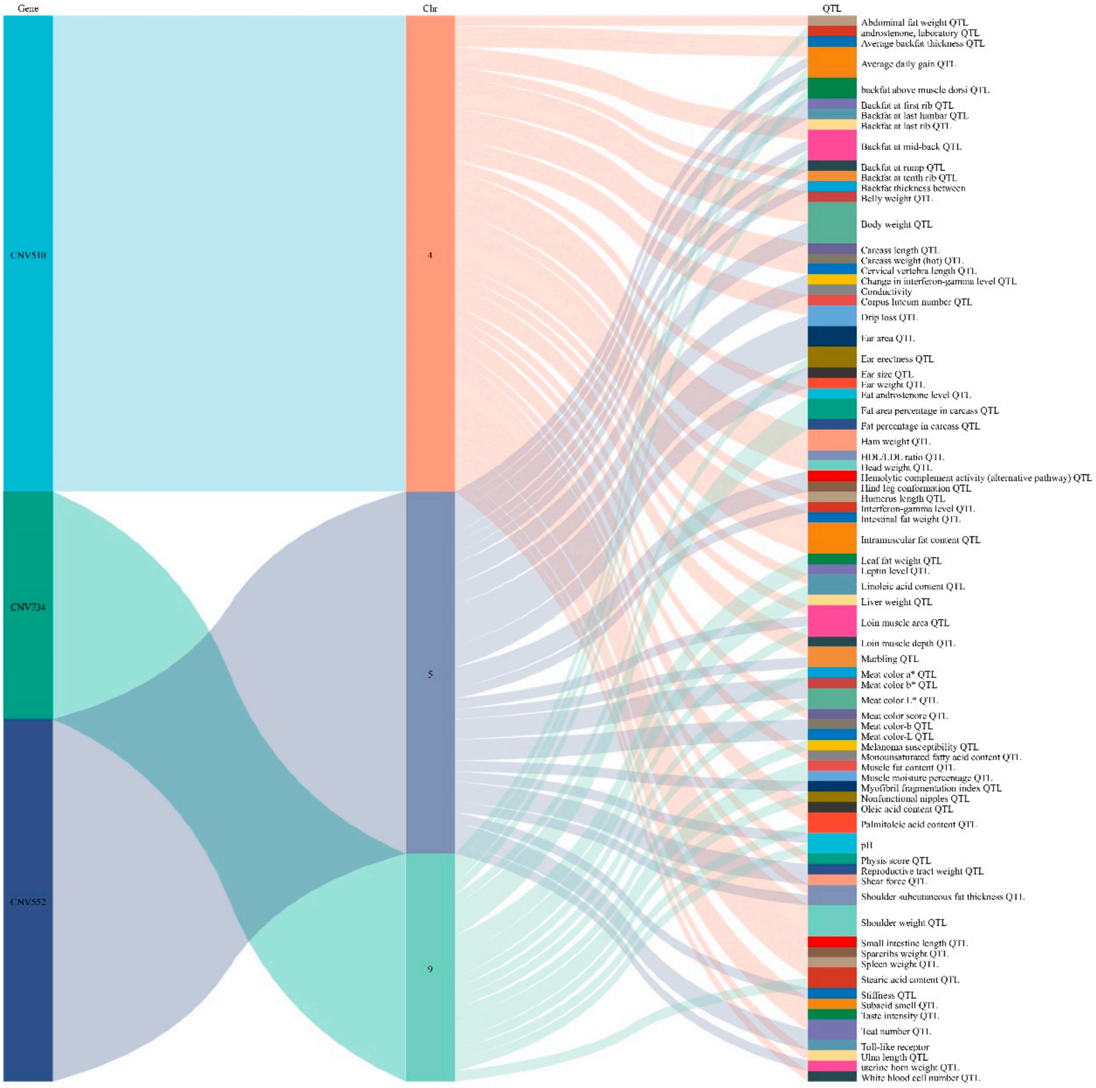

Figure S2: The intersection of CNV 22, CNV267, and CNV390 with swine QTL data

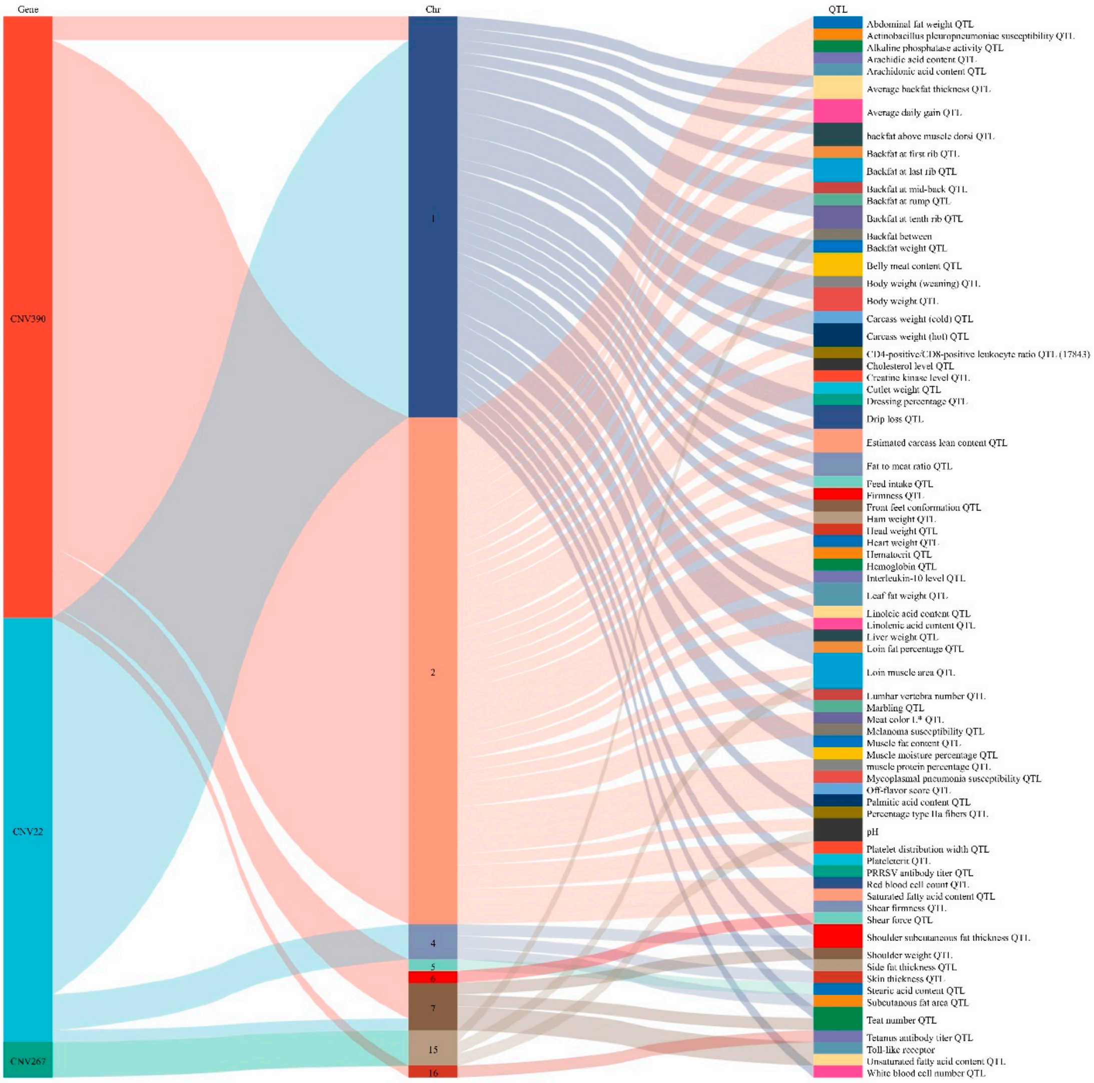

Figure S3: The intersection of CNV321, CNV330, CNV720 and CNV107 with swine QTL data

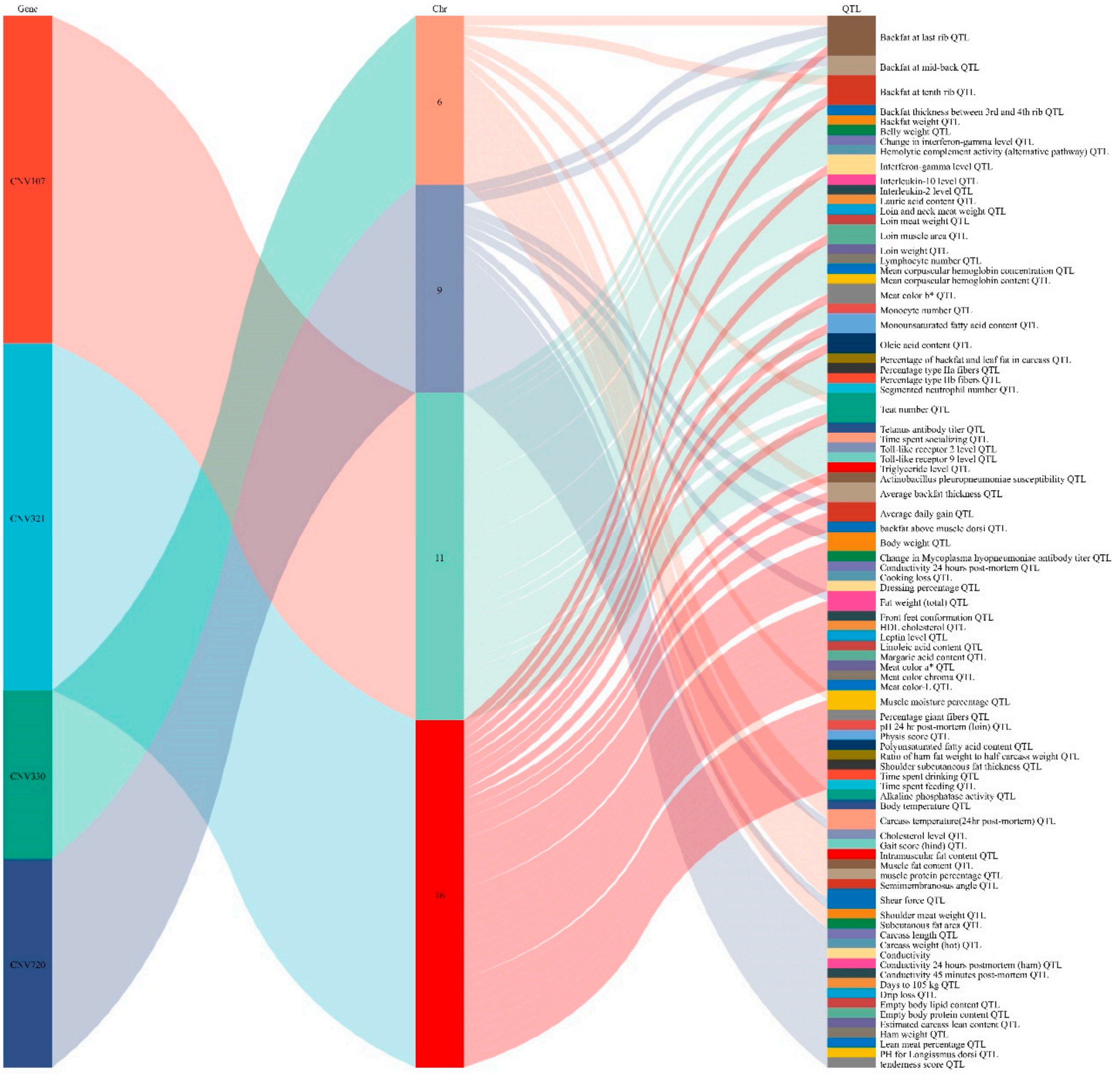

Supplement: Supplementary file 1 [file vetsci-10-00307-s001.zip › vetsci-2307978-supplementary.pdf]
